# Occurrence of *Acanthamoeba* Genotypes in Wastewater Samples in Tehran, Iran

**Published:** 2017

**Authors:** Massoud BEHNIA, Karim HATAM-NAHAVANDI, Elham HAJIALILO, Maryam NIYYATI, Fatemeh TARIGHI, Azam BAKHTIAR AKRAM, Mahboobeh SALIMI, Mostafa REZAEIAN

**Affiliations:** 1. Dept. of Medical Parasitology and Mycology, School of Public Health, Tehran University of Medical Sciences, Tehran, Iran; 2. Infection Disease and Tropical Medicine Research Center, Zahedan University of Medical Sciences, Zahedan, Iran; 3. Dept. of Medical Parasitology and Mycology, School of Medicine, Qazvin University of Medical Sciences, Qazvin, Iran; 4. Dept. of Medical Parasitology and Mycology, School of Medicine, Shahid Beheshti University of Medical Sciences, Tehran, Iran; 5. Center for Research of Endemic Parasites of Iran (CREPI), Tehran University of Medical Sciences, Tehran, Iran

**Keywords:** *Acanthamoeba*, Genotype, Wastewater, Iran

## Abstract

**Background:**

*Acanthamoeba* spp. is potentially pathogenic free-living amoeba that can exist in various water sources. The presence of this amoeba in water sources could be a health hazard as *Acanthamoeba* could lead to severe diseases such as *Acanthamoeba* keratitis and encephalitis. This study aimed to determine the genotypes of isolated *Acanthamoeba* spp. in raw wastewater samples in Tehran, Iran.

**Methods:**

Overall, 90 raw wastewater samples were collected from water treatment facilities in west and south of Tehran, Iran during 2014–2016. Water samples were filtered and cultured on non-nutrient agar (NNA) medium enriched with *Escherichia coli*. Morphological and molecular analyses were done on positive strains. The pathogenic ability of the isolated strains was determined using physical assays.

**Results:**

Totally, 6 out of 90 (6.7%) samples were positive for *Acanthamoeba*, according to morphological characteristics of double-walled cysts. Genotyping and sequencing of the positive strains showed *Acanthamoeba* belonging to T4 (83%) and T11 (17%) genotypes. In vitro pathogenicity tests were revealed that five isolates were classified as non-pathogenic strains and one strain belonging to T4 genotype was classified as the highly pathogenic amoebae.

**Conclusion:**

The current research reflected a low contamination of wastewater sources to *Acanthamoeba*. More studies regarding the contamination of wastewaters before and after treatment are required in different places of the country.

## Introduction

*Acanthamoeba* spp. are one of the most prevalent protozoa with worldwide distribution, these amphizoics amoebae were isolated from various environmental sources such as different kinds of waters including seawater, tap water, aquarium, fresh and saltwater lakes, hot spring resorts, bottled water and swimming pool (1, 2). To date, the classification of *Acanthamoeba* using molecular approaches revealed 20 genotypes (T1–T20) based on 18S rDNA gene region (3), most genotypes have been isolated from clinical cases, however, T4 genotype is the most common strain among the clinical and environmental specimens. The T4 genotype has greater pathogenic ability among the others (1, 4). Moreover, T4 genotype is the most common genotype isolated from corneal scrapes of keratitis patients in Iran (5, 6).

*Acanthamoeba* spp. acting as opportunistic amoebeae is able to develop two severe diseases including Granulomatous Amoebic Encephalitis (GAE) and sight-threatening corneal infection termed *Acanthamoeba* keratitis (AK) (1). The major risk factor for AK is the usage of contact lenses and exposure to contaminated water. Moreover, swimming or bathing in the contaminated aquatic environment is hazardous to contact lens wearers (1, 2). Since *Acanthamoeba* could act as a reservoir for pathogenic microorganism, this could double the threat for human health (1, 7). Several studies in different parts of Iran showed the occurrence of *Acanthamoeba* genotypes and other free-living amoebae (FLA) in the various sources of waters. The presence of *Acanthamoeba* was revealed in recreational water and tap water sources in northern and southern Iran, Gilan and Kish Island, respectively. The results showed the occurrence of T4 genotype in recreational waters and T3, T4, T5 and T11 genotypes in tap water sources (8, 9). Studies on river waters and hot springs in the country were also detected T3, T4, T5 and T15 genotypes of *Acanthamoeba* and *Naegleria* (10–13).

Despite numerous studies conducted on water sources in Iran, there were no data regarding the contamination of wastewater sources to *Acanthamoeba* spp. in the region and thus the main aim of the current study was to determine the occurrence of *Acanthamoeba* genotypes in wastewater of two treatment facilities in west and south of Tehran Province. The pathogenic potential of the isolated strains were also evaluated using thermos and osmotolerance assays.

This study aimed to determine the genotypes of isolated *Acanthamoeba* spp. in raw wastewater samples in Tehran, Iran.

## Materials and Methods

### Sampling

During 2014–2016, 90 raw wastewater samples were collected from water treatment facilities in west and south of Tehran Province. Fifty water samples were collected from the west of the Tehran, Shahrak-e Ekbātān, and 40 samples were obtained from the south of Tehran, Chahardonge region, both areas are located in the urban region. All of the specimens were transferred to Department of Medical Parasitology and Mycology, School of Public Health, Tehran University of Medical Sciences, Iran.

### Sample processing and microscopic survey

Filtration of each sample was performed using nitrocellulose membranes (45-μm diameter). The filters were cultured on 1.5% non-nutrient agar (NNA) medium plates with heat-inactivated *Escherichia coli* (14). The plates monitoring was performed daily using an inverted microscope (15). Cloning of the positive plates was done to purify the amoebae from bacterial and fungal contamination (5).

### In vitro pathogenicity tests

Osmo-tolerance and thermo-tolerance assays were performed on the positive strains. For osmotolerance test, non-nutrient agar with 1 M and 0.5 M mannitol concentration was used to evaluate the outgrowth of amoebae (16). For thermo-tolerance assay, the growth of amoebae was surveyed by the inverted microscopy. The temperature was set at 37 and 42 °C for a week (17). All the plates were monitored for one week.

### DNA extraction, PCR amplification, and Sequencing

*Acanthamoeba* spp. were harvested using sterile phosphate buffered saline (PBS), at pH 7.2, then the harvested amoebae were concentrated and lysed using lysozyme and glass beads treatment. High Pure polymerase chain reaction (PCR) Template Preparation Kit (Roche, Mannheim, Germany) was used for DNA extraction. PCR amplified the ASA.S1 region (500 bp) of 18S rRNA gene, along with primers JDP1 5′-GGCCCAGATCGTTTACCGTGAA-3′ and JDP2 5′-TCTCACAAGCTGCTAGGGAGTCA-3′ (16). A total of 30-μl volume of PCR reaction was fixed by using Amplicone (Taq DNA Polymerase Master Mix RED, Denmark), 0.1 μM of each primer, distilled water, and DNA template. The cycling conditions stared with 94 °C for 1 min; 35 cycles of 94 °C for 35 sec, annealing step was 56 °C for 45 sec and 72 °C for 1 min; final extension was 72 °C for 10 min. The presence of PCR product bands was confirmed with UV light after electrophoresis and staining of PCR product. Purification and sequencing were done using the ABI 3130X sequencer. The sequences adjusted with chromas (ver. 1.0.0.1), afterward compared with BLAST GenBank database.

## Results

**Fig. 1:**
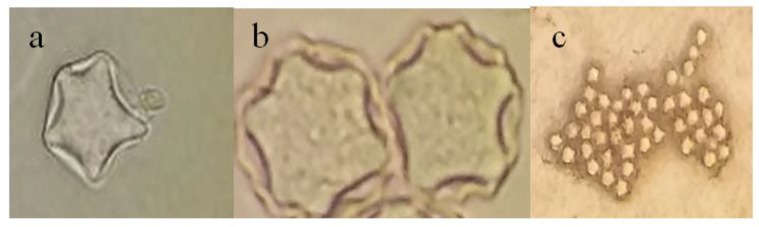
Light microscopy photographs of *Acanthamoeba* cyst in non-nutrient agar. (a) T11 genotype. (b) T4 genotype ×1000. (c) T4 genotype ×100

Of 50 collected samples from the raw wastewater treatment facility of Shahrak-e Ekbatan, 1 (2%) was positive for *Acanthamoeba* spp. From the other 40 specimens belonged to Chahardonge, 5 (12%) were positive (Table 1). Morphologically, all positive strains were belonged to group two morphology (Fig. 1). The result of sequencing showed that 1 (17%) strain labeled as a TWS2 belonged to T11 (corresponding to *A. hatchetii* with 100% homology to the genes available in the gene data bank) and 5 (83%) belonged to T4 genotype (TWS1, TWS3, TWS4, TWS5, TWS6).

**Table 1:** Data of genotyping and pathogenicity test from wastewater samples during 2014–2016, Tehran, Iran

***Code***	***Locality***	***NNA culture***	***PCR***	***Genera***	***Genotype***	***Pathogenicity***
TWS1	Ekbātān	+	+	*Acanthamoeba*	T4	High
TWS2	Ekbātān	+	+	*Acanthamoeba*	T11	Low
TWS3	Ekbātān	+	+	*Acanthamoeba*	T4	Low
TWS4	Ekbātān	+	+	*Acanthamoeba*	T4	Low
TWS5	chahardonge	+	+	*Acanthamoeba*	T4	Low
TWS6	Ekbātān	+	+	*Acanthamoeba*	T4	Low

TWS: Tehran wastewater sample

In vitro pathogenicity tests including osmotolerance and thermo-tolerance assays were confirmed that only a single isolate (TW1) was highly pathogenic as the strain showed a good growth at high temperature and osmolarity, while the other strains were classified as non-pathogenic amoebae (Table 1).

## Discussion

This study is the first survey of *Acanthamoeba* spp*.* isolated from the wastewater specimens in the country. Our study showed 6.7% of the samples were positive for *Acanthamoeba* spp., in which 83% belonged to T4 genotype. The data of this study reflected the occurrence of *Acanthamoeba* spp. in the wastewater of the capital in the country. However, despite the other environmental studies in Iran, the current research reflected a low contamination of wastewater sources to *Acanthamoeba*. This may be due to inhibitory effect of the contaminant in the polluted water. Only *Acanthamoeba* was detected in the studied samples and other free-living amoebae were not observed. This can be explained by more resistance of *Acanthamoeba* cysts in harsh conditions and the fact that other free-living amoebae are more fragile (18). The result of the other studies is opposite to ours. A study conducted in wastewater treatment plant (WWTP) process and sludge in agriculture in the Navarra Community of Spain region showed *Acanthamoeba* spp. as the most common protozoa in outlet water and sludge (19). Moreover, another research in wastewater treatment plant of central Spain detected *Acanthamoeba* belonged to T16 and T7 genotypes. Moreover, phylogenetic analysis detected T19 genotype among the samples (20). Another research in South Africa showed the occurrence of free-living amoebae (87.2%) in wastewater treatment plant (21). The current study showed the presence of T4 and T11 genotypes in the contaminated water samples.

*Acanthamoeba* belonging to T3, T4, T7 and T9 genotypes*, Hartmannella* and *Naegleria* were isolated from the Spanish wastewater treatment plants (22). Moreover, the most frequent FLA was the genus *Acanthamoeba* (59%) from wastewater in the north of Mexico City (23). Despite the mentioned researchers in the world, there were no data regarding *Acanthamoeba* spp. distribution of wastewater samples in Iran.

Most of the wastewaters recovered after treatment and reused directly for irrigation systems, river flow augmentation and industrial consumption (13, 24). As expected, the present study reflected that the most common strain belonged to T4 genotype. T4 genotype was also the frequently isolated strain from clinical and environmental samples (5, 6, 10). However, the interesting point in the present research was the low pathogenicity of most strains. This is in agreement with study that revealed not all T4s have pathogenic potential (25). Moreover, nine strains from nasal swabs of immunocompromised patients belonged to T4 genotype were classified as non-pathogenic ones (16).

*Acanthamoeba* belonged to T11 genotype (*A. hatchetii*) was another isolated strain in the wastewater specimens, this genotype was reported from the keratitis patients in Iran and isolated from the environmental samples previously (6, 26). However, this strain showed low pathogenic potential. More studies regarding the pathogenic ability of the isolated strains such as in vivo tests and animal-based studies are of importance.

## Conclusion

The data of current study was reflected the low occurrence of *Acanthamoeba* genotypes in the raw wastewater samples. More studies about the contamination of wastewaters before and after treatment are required in different places of the country.
